# Superconductivity in the high-pressure tetragonal phase of UTe_2_

**DOI:** 10.1073/pnas.2621369123

**Published:** 2026-10-15

**Authors:** Yuhang Deng, Gabriel Mee, Tyler Wannamaker, Keke Feng, Mingyu Xu, Weiwei Xie, M. Brian Maple

**Affiliations:** ^a^ Department of Physics, University of California San Diego, La Jolla, CA 92903; ^b^ Department of Chemistry, Michigan State University, East Lansing, MI 48824

**Keywords:** UTe_2_, phase transition, high pressure, spin-triplet superconductor, heavy-fermion compound

## Abstract

Electrical transport and magnetic measurements have been made on UTe_2_ under pressure *P* up to ~16 GPa to determine the superconducting transition temperature *T*_c_ vs. *P* phase diagram in the high-pressure tetragonal phase. Superconductivity emerges near ~5 GPa, coincident with the orthorhombic—tetragonal phase transition; in the tetragonal phase, *T*_c_ reaches a maximum value of ~4 K at 6 GPa and then decreases with *P* and appears to vanish near 18 GPa. Tetragonal UTe_2_ has a relatively small upper critical field *H*_c2_(0) ≈ 1.2 T at 5.3 GPa, smaller than the Pauli paramagnetic limit, and is orbitally limited with a coherence length ξ_tetra_ ≈ 16.5 nm. This small value of *H*_c2_(0) favors more conventional superconductivity; in contrast, the large values of *H*_c2_(*T*) for orthorhombic UTe_2_ exceed the Pauli paramagnetic limit in all three crystallographic directions and have been attributed to unconventional superconductivity, widely believed to involve spin-triplet pairing. The temperature–pressure phase diagram of UTe_2_ shows a striking dichotomy: a narrow, fragile, unconventional superconducting region in the orthorhombic phase vs. a broad, robust, and more conventional superconducting dome in the tetragonal phase. This dichotomy is consistent with the proposal that U-dimers, present (absent) in the orthorhombic (tetragonal) phase, may play a role in spin-triplet superconductivity of orthorhombic UTe_2_. In the tetragonal phase, the normal-state electrical resistivity ρ(*T*) exhibits metallic behavior with a “knee” at 200 to 240 K that depends weakly on *P* and most likely marks the onset of a transition to a magnetically ordered phase that coexists with superconductivity.

## Significance

Understanding the origin and nature of conventional and unconventional superconductivity remains a central challenge in strongly correlated electron physics. In UTe_2_, we mapped out the temperature vs. pressure phase diagram of a more conventional superconducting state hosted in the high-pressure tetragonal phase that exhibits a broad superconducting dome, small upper critical fields, and weaker electronic correlations compared with the unconventional superconductivity in orthorhombic UTe_2_. The striking contrast between the two phases suggests that structural changes and uranium bonding configurations strongly influence the superconducting pairing mechanism and the underlying electronic correlations. Our results establish UTe_2_ as a rare platform for exploring the interrelation between distinct superconducting regimes tuned by pressure within a single material.

The recently discovered heavy-fermion compound uranium ditelluride (UTe_2_) with *T*_c_ = 2.1 K has attracted intense interest due to its extraordinary superconducting properties at ambient pressure (1, 2), magnetic field reentrant and field polarized superconductivity (SC_FP_) (3–6), and complex interplay between ferromagnetic spin fluctuations (7, 8), antiferromagnetic (AFM) spin fluctuations (9–13) and unconventional superconductivity. Its potential realization of spin-triplet, chiral, odd-parity superconductivity positions UTe_2_ as a strong candidate for topological superconductivity, offering promising avenues for quantum technologies (1, 14–18). However, despite extensive studies, a comprehensive understanding of its superconducting and magnetic properties, electronic states, and crystal structures remains elusive, especially under high pressure where limited experimental probes could be applied.

Under applied pressure (*P*) below 4 GPa and in high magnetic fields, UTe_2_ undergoes a series of electronic and magnetic transitions. At ambient pressure and in high magnetic field (*H*), UTe_2_ has three superconducting phases: SC_1_ below ~20 T, SC_HF_ between 20 and 35 T near the *b*-axis, and SC_FP_ above 35 T that exists in a narrow angular region in the *bc* plane inside the field polarized region (19). When *P* > 0.3 GPa, another superconducting phase, the high-pressure-zero-field phase SC_2_, appears and coexists with SC_1_ with a higher *T*_c_ (20, 21). It was later concluded that the SC_2_ and SC_HF_ phases have the same identity by means of specific heat measurements as a function of *H*, *P,* and *T* which show that the two phases evolve continuously into one another (22). Under higher pressure (*P* ≳ 1.7 GPa), SC_1_ and SC_2_ are suppressed and replaced by long-range AFM order, revealed by neutron diffraction (23), AC calorimetry, and electrical transport measurements (21). The emergence of this AFM order is likely due to the pressure-enhanced AFM fluctuations near *P*_c_ (23) and seems to occur simultaneously with a U valence change toward U^4+^ (21, 24).

When *P* is higher than ~4 GPa, UTe_2_ undergoes a transition from an orthorhombic (space group *Immm*) to a higher-symmetry tetragonal crystal structure (*I*4/*mmm*), accompanied by a sudden volume collapse with an increase in the nearest U-U separation (25, 26) and a change in the uranium electronic configuration toward a larger fraction of the 5*f* ^3^ configuration (24, 27) revealed by x-ray spectroscopies. In the tetragonal phase, the electrical transport behavior of UTe_2_ is characterized by a knee at T^**^ ≈ 230 K in electrical resistivity (ρ) vs. *T* curves (26), which was suggested to represent a crossover from localized 5*f*-electron behavior to a coherent array of 5*f*-electrons (26) or a transition to a magnetically ordered state (28). Moreover, bordering the AFM phase at lower pressures in the orthorhombic phase, a different superconducting region emerges in the tetragonal phase with weaker electronic correlations and smaller upper critical fields (*H*_c2_), revealing a type of superconductivity that is distinctly different from the low-pressure heavy-fermion unconventional superconductivity (26). Interestingly, it was claimed that possible metamagnetic transitions appear in tetragonal UTe_2_ in high magnetic fields applied nearly along the *c*′-axis of the tetragonal phase, or tilted at 30° from the *b*-axis toward the *c*-axis of the orthorhombic phase (28), exactly where the orthorhombic SC_FP_ phase is accommodated after the metamagnetic transition induced by high fields. Accessing pressures up to 16 GPa, we combined electrical resistivity, AC magnetic susceptibility, and structural insights to track the evolution of the properties of UTe_2_ across the orthorhombic–tetragonal structural transition and uncover the more complete superconducting phase diagram of the tetragonal phase of UTe_2_.

## Results

**Fig. 1.**
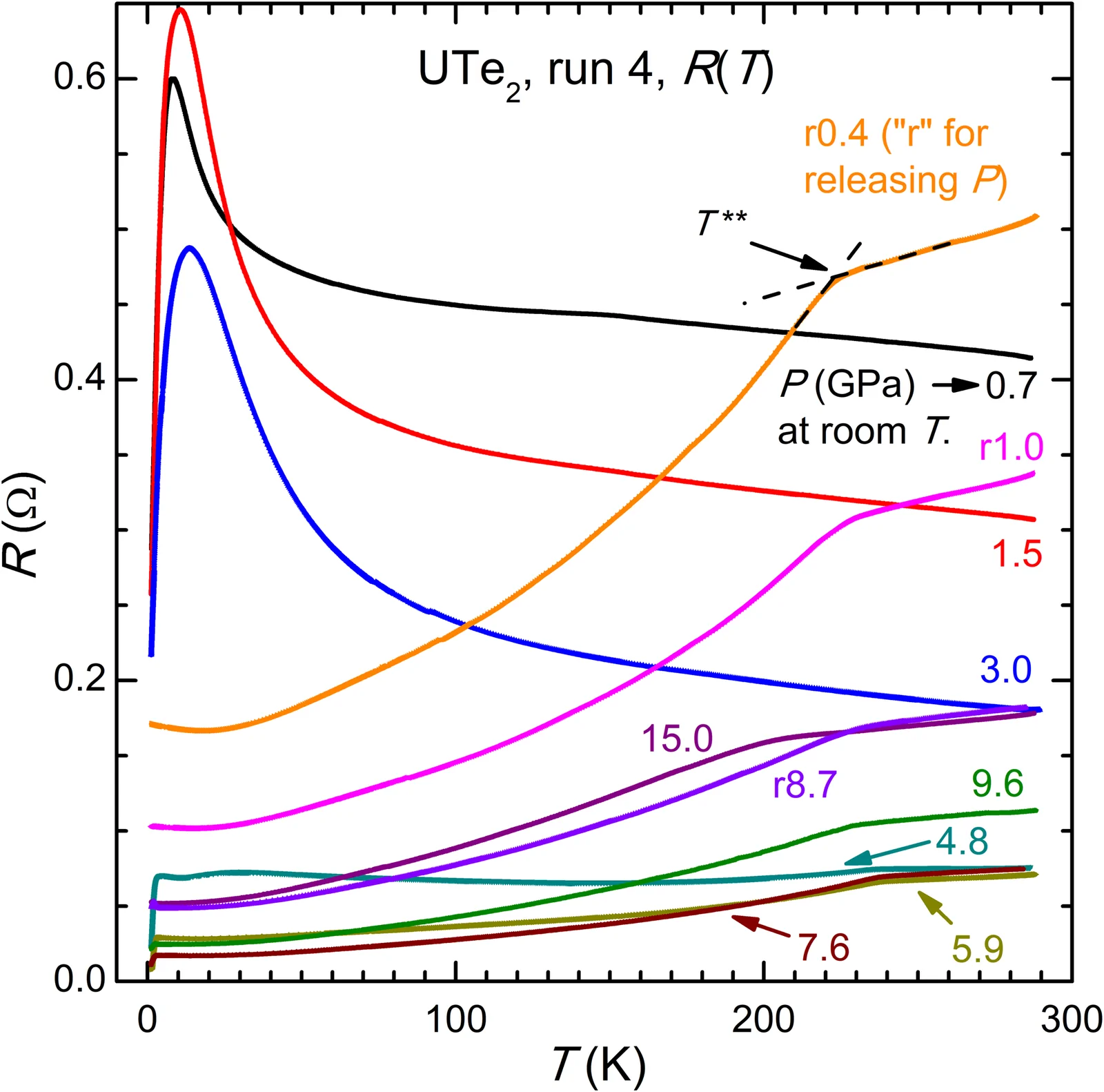
Representative *R*(*T*) curves for UTe_2_ measured at different pressures in Run 4. The values of pressure shown were determined at room temperature. The letter “r” before some of the numbers refers to measurements taken upon release of pressure from the highest pressure of 15 GPa.

The two AC χ(*T*, *P*) runs revealed a diamagnetic signal at ~1.9 K below 1 GPa from the superconducting transition in orthorhombic UTe_2_ at low pressures as seen in *SI Appendix*, Fig. S2 in *SI Appendix*, but were not able to detect an obvious transition in the pressure region where UTe_2_ has the tetragonal structure. However, *R*(*T*) data at high pressure, as presented in Fig. 1, clearly demonstrate dramatic changes in electrical transport and the superconducting transition above ~4 GPa where UTe_2_ transforms into the tetragonal phase. We also noted that for pressures near this transition, the cooling *R*(*T*) curves deviate from the warming ones above ~80 K, which is likely because UTe_2_ is undergoing the orthorhombic-to-tetragonal transition promoted by the increase in pressure that occurs in the DAC upon cooling. Before the structural transition, *R*(*T*) in the normal state exhibits Kondo-like behavior at high *T* with a pronounced peak at ~10 K; after the transition, the *R*(*T*) curves are dramatically different than those before the transition and feature metallic behavior (d*R*(*T*)/d*T* > 0) with a knee at T^**^ ≈ 235 K which we define as the temperature where the two extrapolated linear regions near the knee intersect. The curve at ~4.8 GPa near the boundary of the structural transition shows a dual nature in which the peak in *R* (orthorhombic feature) and the knee in *R* accompanied by the superconducting transition (tetragonal features) coexist. Interestingly, during release of pressure, *R*(*T*) at 1.0 and 0.4 GPa retains the knee but there is no drop in resistance due to the superconducting transition, indicating the metastable tetragonal phase that persists to nearly ambient pressure does not exhibit superconductivity above 1.2 K.

**Fig. 2.**
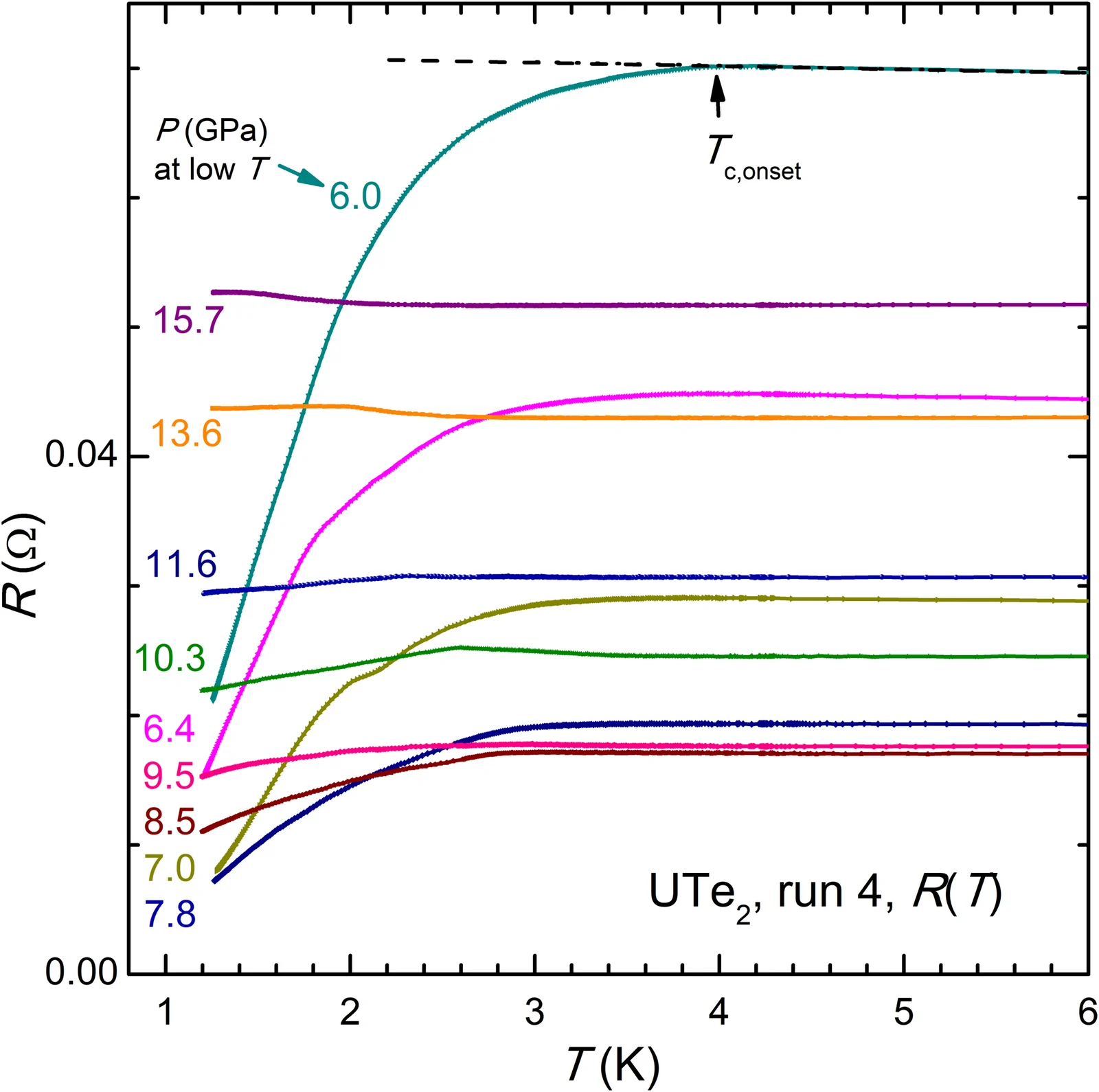
Resistive superconducting transition curves of the tetragonal UTe_2_ sample studied in Run 4. The *R*(*T*) data are for pressures calibrated at temperatures near the superconducting transitions. *T*_c,onset_ is defined as the temperature where the *R*(*T*) curve starts to deviate from the linear extrapolation of *R*(*T*) in the normal state.

To more clearly display the superconducting transition of tetragonal UTe_2_, Fig. 2 shows *R*(*T*) curves in the range 1 K to 6 K for *P* > 4 GPa. For most of the curves, a clear resistance drop indicates the occurrence of a superconducting transition which confirms the report by Honda et al. (26), although zero resistance could not be achieved due to the low temperature limit of the experiments and, possibly, pressure inhomogeneity (stress) across the sample manifested in the broad transition width. Above 12 GPa, an unexpected resistance increase instead of decrease with cooling was observed, which could be attributed to the redistribution of electrical current that often appears during superconducting transitions in inhomogeneous pressurized samples. As a result, we propose that tetragonal UTe_2_ is still superconducting with a *T*_c_ above 1 K to the highest pressure we investigated, namely about 16 GPa.

**Fig. 3.**
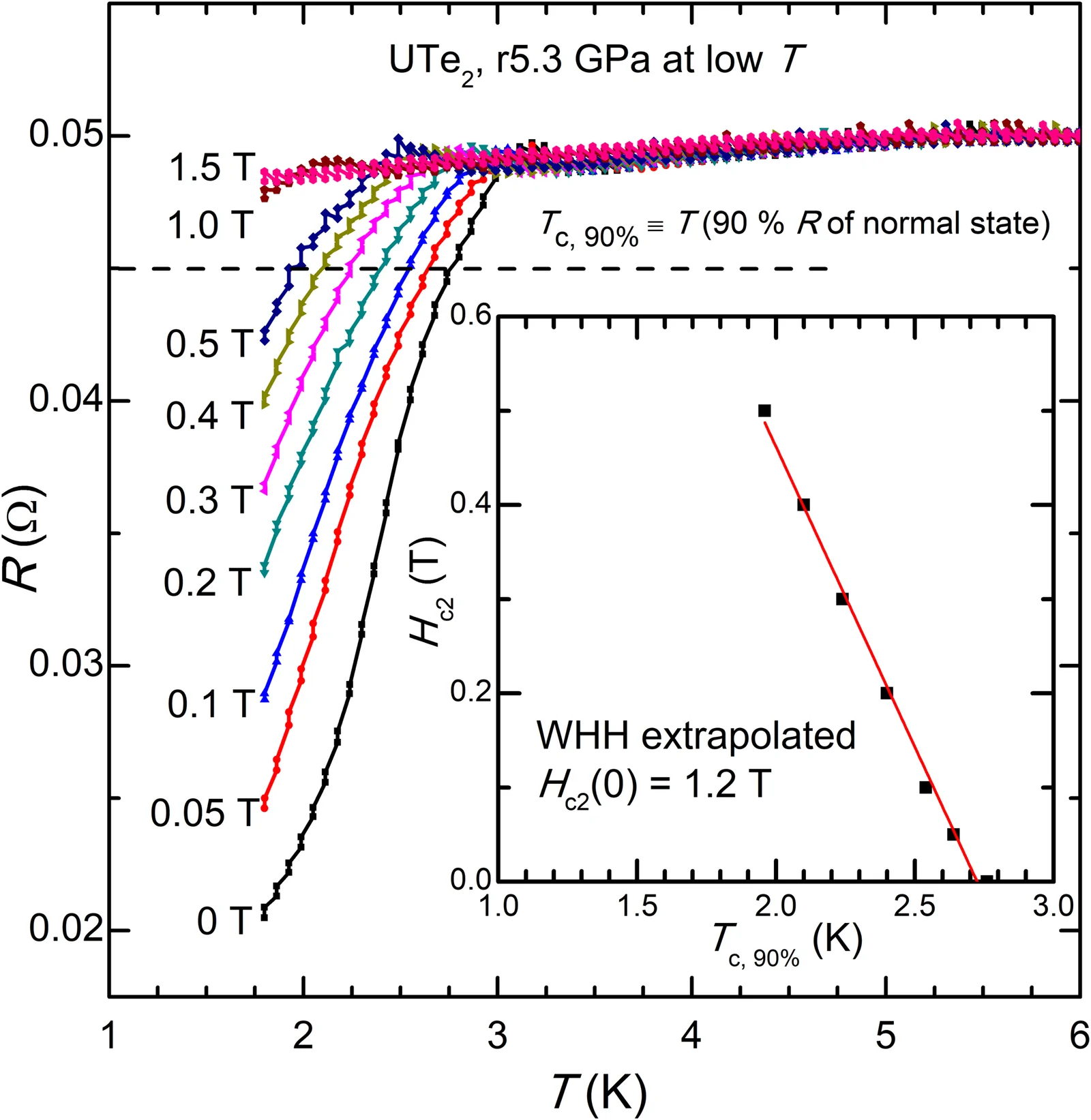
*R*(*T*) curves measured in magnetic fields from 0 T to 1.5 T at *P* = r5.3 GPa. *T*_c, 90%_ was determined from the temperature at which *R* drops to 90% of its value in the normal state. The *Inset* shows a plot of the upper critical field *H*_c2_ vs. *T*_c,90%_ in low fields. The red straight line is a linear fit to the data which was used to estimate *H*_c2_(0) by means of the Werthamer–Helfand–Hohenberg (WHH) theory.

Magnetic fields were applied to the tetragonal sample to study the upper critical field (*H*_c2_) of UTe_2_ at r5.3 GPa. As shown in Fig. 3, a field as low as 1 T almost completely suppressed the superconducting transition. Based on the Werthamer–Helfand–Hohenberg (WHH) theory, *H*_c2_(0) due to the orbital pair-breaking mechanism in the dirty limit can be estimated using the equation:

[1] $$H_{c2}\left(0\right)\;=\;-0.693T_{c}\left(0\right)\frac{dH_{c2}}{dT_{c}}{|}_{T_{c}(0)}.$$

We obtain *H*_c2_(0) ≈ 1.2 T for tetragonal UTe_2_ at r5.3 GPa. In contrast, for UTe_2_ in the orthorhombic structure, the smallest *H*_c2_(0) which is along the magnetic easy axis (a-axis) is about 5 T (1), and even larger (10 T) (19) in UTe_2_ with enhanced superconductivity that was synthesized by a similar method as our sample. On the other hand, *H*_c2_(0) dominated by the paramagnetic pair-breaking mechanism (Pauli limit) can be calculated using the equation:

[2] $$H_{\mathrm{Pauli}}\;=\;1.84\;T_{c}(0).$$

Using the data at r5.3 GPa, the Pauli limit is ~5.1 T, significantly larger than the orbital limit, which suggests the superconductivity is mainly orbitally limited, different from the situation for UTe_2_ at ambient pressure (in the orthorhombic phase). The Ginzburg–Landau coherence length ξ can be estimated from the relation:

[3] $$\mu_{0}H_{c2}\left(0\right)\;=\;\Phi_{0}/2\pi \xi^{2},$$

where Φ_0_ is the magnetic flux quantum, which yields for tetragonal UTe_2_ the value ξ_tetra_ ≈ 16.5 nm. Note that the orientation of the pressurized UTe_2_ samples was not determined so this value is not specified for a certain axis of tetragonal UTe_2_.

**Fig. 4.**
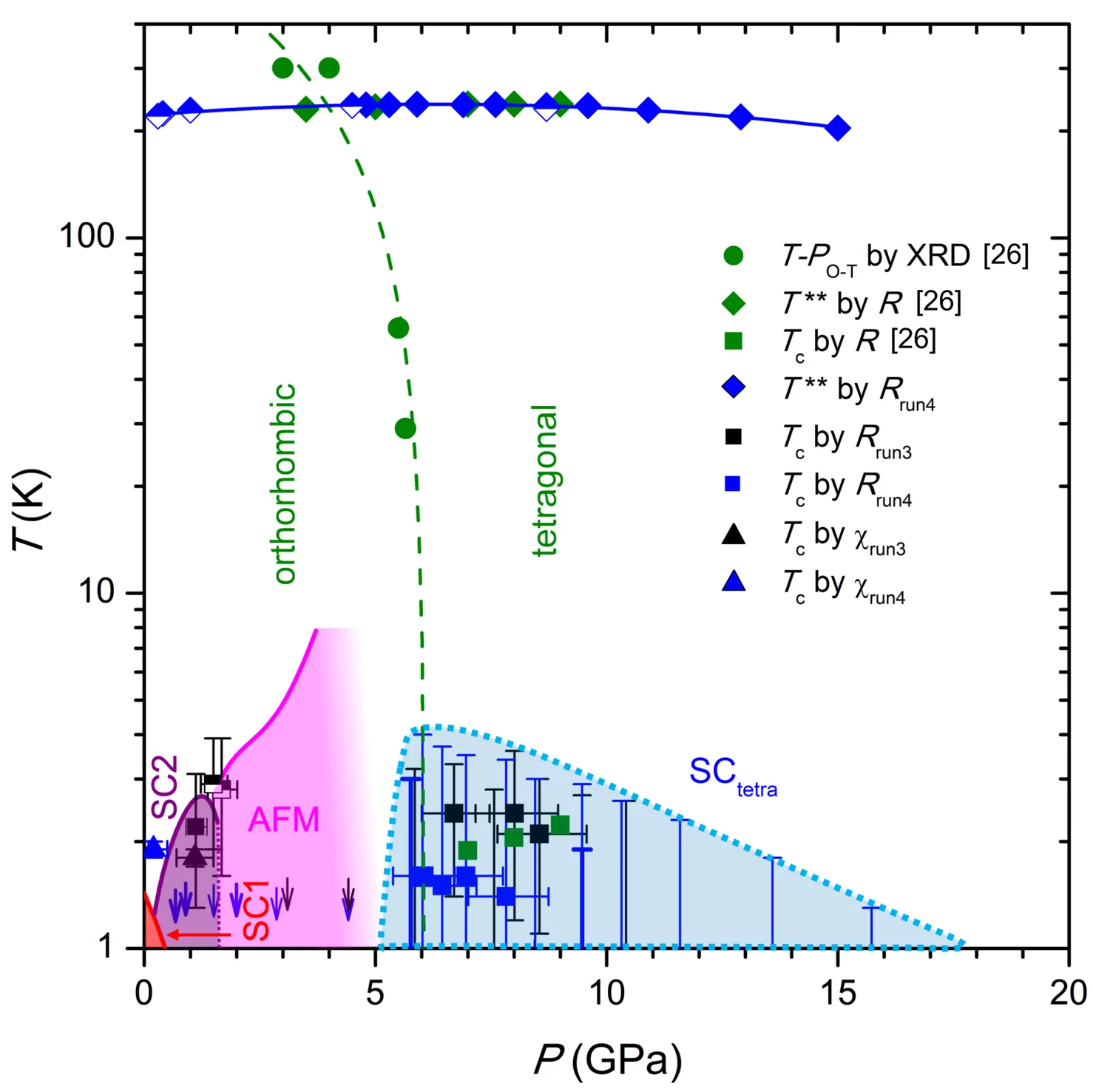
*T*–*P* phase diagram for UTe_2_. For *R*(*T*, *P*) measurements, *T*_c,zero_ (lower error bar) is determined by extrapolating the linear region to *R* = 0 Ω. *T*_c,mid_ (symbols) is determined by finding the temperature at the midpoint between the resistances at *T*_c,onset_ (upper end of error bar) and *T*_c,zero_. For some curves, the extrapolated *T*_c,zero_ < 0 K, so *T*_c,zero_ and *T*_c,mid_ could not be determined. Down pointing arrows indicate no superconductivity was found down to *T* at the point of the arrow. For AC χ(*T*, *P*) measurements, the triangular symbols and their error bars stand for the midpoints and the onset/end point temperatures of the transitions, respectively. The dashed line through the solid green circles (26) separates the orthorhombic and tetragonal structure regions. The orthorhombic UTe_2_ superconductivity regions (SC_1,2_) and antiferromagnetic (AFM) region are depicted based on data reported in refs. 21, 23, and 26.

A *T*–*P* phase diagram was constructed as shown in Fig. 4 that reveals the evolution of superconductivity and the knee in *R*(*T*) at T^**^ in tetragonal UTe_2_ with applied pressure—the green points are taken from Honda et al. (26) and the closed points (collected during increasing *P*) and half-closed points/bold face upper error bars (collected during releasing *P*) from our *R*(*T, P*) and AC χ(*T*, *P*) measurements. Blue shading indicates the superconducting *T*–*P* region which mainly resides within the tetragonal phase of UTe_2_. The small shaded region to the left of the green line delineating the orthorhombic-tetragonal phase boundary probably belongs in the tetragonal region and is displaced due to a small differences in pressure scales between our experiments and those of Honda et al. (26), unavoidable pressure gradients in our DAC experiments, uncertainty in determining the low-*T* pressure, and the sluggish nature of the structural transition (25, 27). As can be seen, the tetragonal superconductivity (SC_tetra_) could extend to ~18 GPa, taking a “half dome” shape with a broad maximum near the orthorhombic-tetragonal phase boundary.

The resistance knee is an important feature of the tetragonal phase of UTe_2_ and appears simultaneously with the tetragonal phase superconductivity. The knee feature is very robust and persists to the highest pressure we investigated: T^**^(*P*) increases slightly from 237 K at 4.8 GPa to 238 K at 5.9 GPa and then decreases slowly with pressure to 204 K at 15 GPa. There may be a correlation between T^**^ and *T*_c_ in the tetragonal phase of UTe_2_, namely, the maximum T^**^ is at pressures where *T*_c_ reaches nearly its highest value. This correlation was also observed in ref. 26 where more hydrostatic pressure was applied. Although T^**^ becomes a little smaller with decreasing *P*, the resistance knee remains down to nearly ambient pressure, which again shows that the knee is an electronic hallmark of the tetragonal phase as our high-pressure XRD results in *SI Appendix* indicated that the tetragonal phase is retained at least down to 1.4 GPa.

## Discussion

### The Possible Origin of the Transition at T^**^.

As discussed above, the resistance knee at T^**^ seems to be a hallmark of the electrical transport of tetragonal UTe_2_. It resembles the knee features at T^*^ ≈ 50 K in the *R*(*T*) curves of UTe_2_ along the *a* and *b* axes at ambient pressure (1, 29) which are usually attributed to a transition from single-ion Kondo behavior to a coherent heavy-fermion ground state, but it occurs at a significantly higher temperature (about 4 to 5 times higher). There are some other reports concerning the T^**^ transition (26, 28, 30) in which a Bridgman anvil cell or a cubic anvil cell was used, providing a more hydrostatic pressure environment than our diamond anvil cell, suggesting the transition is not solely due to the effect of strain. On the other hand, it was found that this transition appeared when the *b*-axis of an orthorhombic UTe_2_ crystal was parallel to the direction of applied pressure but was absent for a sample with the *c*-axis aligned along the direction of applied pressure (30), revealing the role of uniaxial pressure in inducing the transition. If the resistance knee signals the transition to a coherent ground state, it indicates that the Kondo interaction is stronger in tetragonal UTe_2_ than in orthorhombic UTe_2_ and thus enters the coherent ground state at a much higher T^**^ than in orthorhombic UTe_2_. The stronger Kondo interaction indicates larger hybridization between the U-5*f*- and conduction-electron states and a smaller conduction electron effective mass m^*^, consistent with a smaller coefficient of the Fermi liquid contribution to the electrical resistivity *A* = (ρ – ρ_o_)/*T*^2^ (ρ_o_ is the residual resistivity) at low temperatures *T* ≪ T^**^, which is about 1000th of that of the orthorhombic phase (26).

Before the establishment of a coherent ground state, the electrical resistivity ρ(*T*) should have a contribution due to incoherent Kondo scattering that varies as ~–ln*T* and increases in magnitude upon cooling. This contribution was not evident in our measurements (orientation of measured UTe_2_ not determined) or those reported in refs. 26 and 28 (resistance measured along the *a*-axis of the orthorhombic sample). However, it should be noted that the –ln*T* contribution could be difficult to observe because it is based on a single ion theory and could be modified by interactions between U ions, uncertainty in determining the Kondo temperature, and difficulties in separating it from the lattice contribution to ρ(*T*). An alternate interpretation of the apparent transition at T^**^ was proposed by Thebault et al. (28). These authors measured the electrical resistivity of UTe_2_ in the tetragonal phase in strong magnetic fields and observed step functions in the ρ(*H*) curves at *H*_x1_ and *H*_x2_, only at temperatures below T^**^, which lead the authors to propose that T^**^ could be the temperature of a magnetic phase transition below which the spin disorder contribution to electron scattering decreases, and *H*_x1,2_ could be the signatures of some metamagnetic transitions in magnetically ordered tetragonal UTe_2_ (28). According to high-pressure XRD results (25, 27), below 30 GPa, the nearest U-U distance in the tetragonal phase, *d*_U-U_, is larger than the Hill limit (~3.5 Å) (31) for U compounds, favoring a more localized U-5*f* electron configuration and thus a magnetically ordered state (32). From this point of view, it is natural to expect tetragonal UTe_2_ to undergo a transition to some kind of magnetically ordered state.

Although current data do not allow us to settle the debate on the origin of the transition at T^**^, this transition is certainly associated with SC_tetra_: in a certain pressure range, formation of a coherent ground state or some type of order, probably magnetic, and SC_tetra_ coexist down to low temperatures. On the other hand, upon releasing pressure to nearly ambient pressure, even though the measured T^**^ remains similar to that observed at high pressures, no SC_tetra_ was detected above 1.3 K. This decoupling indicates the transition at T^**^ has an electronic origin that is weakly dependent on changes in lattice parameters in the tetragonal phase, in contrast to SC_tetra_, which is more sensitive to lattice parameter changes.

### Conventional or Unconventional Superconductivity in Tetragonal UTe_2_.

We obtained ξ_tetra_ ≈ 16.5 nm for the Ginzburg–Landau coherence length using Eq. **3**. Orthorhombic UTe_2_ has high magnetic anisotropy and the coherence lengths at ambient pressure were reported to vary from 2.4 to 15 nm (15, 33, 34) depending on the direction of the applied magnetic field and measurement methods. The larger ξ_tetra_ and the relation *H*_c2_(0) < *H*_Pauli_ indicate SC_tetra_ is more conventional than its orthorhombic counterpart (SC_ortho_). Moreover, the increased symmetry and enlarged U nearest neighbor distance *d*_U-U_ in the tetragonal phase (27) imply reduced 5*f* electron participation in bonding, suggesting a crossover toward a more itinerant electronic state, potentially favorable to more conventional BCS-like superconductivity. It is noteworthy that the element tellurium (Te) at 4.5 GPa has a *T*_c_ of 4 K, *H*_c2_(0) of ~0.3 T, and coherence length of ~34 nm (35). The large difference in *H*_c2_(0) between Te and UTe_2_ at similar pressures indicates that the observed tetragonal superconductivity was not from decomposition of UTe_2_ into U and Te during compression/decompression, which was found in ref. 25.

The orthorhombic phase of UTe_2_ contains a network of two-leg ladders extending in the *a*-direction in which pairs of U atoms, or U dimers with the shortest U-U distance, form the rungs of ladders which are oriented in the *c*-direction. Based on elastic neutron scattering measurements, there is evidence for FM exchange interactions between the U ions in the dimers that form the rungs of the ladder and AFM exchange interactions between U atoms in neighboring ladders (10). The FM interactions between U ions within a U dimer have been considered as possible mechanisms for spin-triplet pairing in UTe_2_ (17, 36). Chen et al. (36) have proposed that AFM exchange interactions could account for the incommensurate AFM fluctuations observed in inelastic neutron scattering experiments (9, 10, 11, 13) and the resonance in the spin spectrum of UTe_2_ (11, 12), as well as the incommensurate AFM ordering under pressure (23). In contrast, there are no U-dimers in the tetragonal phase of UTe_2_, which could account for the fact that it exhibits a more conventional type of superconductivity, as noted above.

The discovery of SC_tetra_ places U-based superconductivity in a broader and more intriguing context. Canonical uranium heavy-fermion superconductors, such as orthorhombic UTe_2_, UPt_3_, UBe_13_, and URu_2_Si_2_ (37), with relatively low *T*_c_s ≲ 2 K, have a *d*_U-U_ that typically exceeds the Hill limit (32), and exhibit strongly correlated electron behavior and unconventional superconducting electron pairing. In contrast, some other uranium superconductors with a smaller Sommerfeld coefficient γ, including α-U under pressure (38, 39) and β-U (40), have a *d*_U-U_ that usually falls below the Hill limit (32), displaying relatively more BCS-like superconductivity. Strikingly, tetragonal UTe_2_ violates this empirical dichotomy: It has the highest value of *T*_c_ (~4 K) among all known U-based intermetallic superconductors even though its *d*_U-U_ exceeds the Hill limit, thus SC_tetra_ seems to belong to a conventional category based on the previous discussion of its structural symmetry, *H*_c2_(0), and *A* coefficient of *R*(*T*) in the normal state. Tetragonal UTe_2_ constitutes a rare scenario in which a U-compound with a large spacing between U ions potentially harbors BCS-like superconductivity instead of unconventional superconductivity or 5*f* electron magnetic ordering, placing UTe_2_ in a previously unexplored regime where structural, electronic, and pairing tendencies are fundamentally at odds with established trends.

We have established the *P*–*T* phase diagram for UTe_2_ through measurements that extend to substantially higher pressures than those reported in ref. 26, revealing the complete superconducting region within the tetragonal phase of UTe_2_ which extends up to at least 30 GPa (27). The resulting phase diagram exposes a striking dichotomy: a narrow, fragile, unconventional superconducting region associated with the orthorhombic phase, in sharp contrast to a wide, robust, potentially conventional superconducting dome stabilized in the tetragonal phase. Below ~5 GPa, multiple kinds of order, including SC_1_, SC_2_, and AFM, coexist or compete (21, 23), whereas above this threshold, SC_tetra_ rapidly dominates and remains relatively stable over an extended pressure range. These observations raise a central and unresolved question: can pressure tune between BCS-like and unconventional superconducting regimes within a single material, like UTe_2_? If so, UTe_2_ would constitute a rare platform for directly interrogating the microscopic evolution between distinct pairing mechanisms though the application of pressure. Our results therefore position UTe_2_ as a critical bridge connecting conventional and unconventional superconductivity, offering a route to unify these seemingly disparate regimes within a single correlated electron system.

## Concluding Remarks

In summary, through electrical transport and AC magnetic susceptibility measurements, we have constructed the *P*–*T* phase diagram of UTe_2_ and identified distinct superconducting regions associated with the orthorhombic and tetragonal phases. Superconductivity in the tetragonal phase is relatively robust with respect to the application of pressure and exhibits a lower upper critical field and larger coherence length, consistent with more conventional superconductivity with orbitally limited behavior. These results suggest a crossover toward a more weakly correlated superconducting state involving fewer 5*f*-electrons under pressure, highlighting the strong connection between structure, electronic correlations, and superconductivity in the UTe_2_ system, that can serve as a testing ground for pressure tuned superconducting mechanisms.

## Materials and Methods

Single crystals of UTe_2_ were synthesized by chemical vapor transport (CVT) (1) or molten salt flux liquid transport (MSFLT) (41). No impurity phases were detected according to powder X-ray diffraction (XRD) using a Rigaku MiniFlex600 diffractometer. Superconductivity at ambient conditions was confirmed by specific heat and electrical resistivity measurements. The MSFLT-grown UTe_2_ single crystals have a higher *T*_c_ (~2.1 K) compared to the CVT-grown crystals with a *T*_c_ of ~2.0 K, consistent with a previous report by Aoki (41). High-pressure XRD measurements were conducted to study the crystal structure of UTe_2_ upon increasing and decreasing pressure, with in-depth technical descriptions in *SI Appendix*.

Diamond anvil cells (DACs) from Almax easyLab and DAC Tools were used to measure the electrical resistance (*R*) and AC magnetic susceptibility (χ), respectively, of UTe_2_ under high pressure. Several measurements were made, among which Run 3 and Run 4 yielded good data. In Runs 3 and 4, the resistance of the UTe_2_ sample was measured under pressure using steatite as the pressure transmitting medium (PTM) by means of the van der Pauw 4-point method. Part way through Run 3, one of the electrical leads lost contact with the sample, so a 3-point configuration was used for measurements at some pressures. For AC χ(*T*) runs, hardened CuBe gaskets were used to minimize the magnetic background, a mixture of Fluorinert 77:70 = 1:1 was employed as the PTM, and the flux expelled by the superconductor was detected using a mutual inductance mini coil with a primary and two oppositely wound secondary coils, one of which contained the sample. The fluorescence of ruby was measured to determine the room-temperature pressure of the UTe_2_ sample. Additionally, a flake of lead (Pb) was placed along with the sample in the AC χ(*T*) experiments to estimate the pressure from the known *T*_c_ of Pb (42). For the *R* measurements, pressures at low temperature were calibrated with *T*_c_ of Pb measured in separate experiments. A customized ^4^He cryostat was used to reach temperatures as low as ~1.2 K by pumping on liquid helium with a Stokes pump. A Physical Property Measurement System (PPMS) DynaCool provided magnetic fields for magnetoresistance measurements.

## Supplementary Material

Appendix 01 (PDF)

## Data Availability

AC magnetic susceptibility, electrical resistivity, X-ray diffraction, ruby fluorescence data have been deposited in Open Science Framework (https://osf.io/agb5m/files/osfstorage). Previously published data were used for this work (21, 23, 26). All other data are included in the manuscript and/or *SI Appendix*.
