# Supplementary material for: Superconductivity in the high-pressure tetragonal phase of UTe2
Source: Proc Natl Acad Sci U S A. 2026 Oct 15;123(42):e2621369123. doi: 10.1073/pnas.2621369123 (PMC13684043; doi:10.1073/pnas.2621369123)
Supplement: Supplementary file 1 [file pnas.2621369123.sapp.pdf]

Supporting Information for
Superconductivity in the high-pressure tetragonal phase of UTe_2

Yuhang Deng[1], Gabriel Mee[1], Tyler Wannamaker[1], Keke Feng[1],
Mingyu Xu[2], Weiwei Xie[2], M. Brian Maple[1,*]

[1]Department of Physics, University of California San Diego, La Jolla, California 92903, USA

[2]Department of Chemistry, Michigan State University, East Lansing, Michigan 48824, USA

[*]Corresponding author: M. Brian Maple, mbmaple@ucsd.edu

Ambient Pressure Single Crystal X-ray diffraction (SCXRD)

A UTe_2 single crystal ($0.14 \times 0.03 \times 0.04$ mm^3) was selected and mounted on a nylon loop with Paratone oil. The measurement was taken at room temperature using a Rigaku XtaLAB Synergy Dualflex diffractometer with a HyPix detector. Data were collected using ω scans with Mo Kα radiation (λ = 0.71073 Å) from a micro-focus sealed tube (50 kV, 1 mA). Data collections were using hemisphere symmetry strategy which were optimized using CrysAlisPro (v1.171.43.144a). Data reduction included Lorentz and polarization corrections, numerical absorption correction based on Gaussian integration over a multifaceted crystal model[1], and empirical spherical harmonics correction using the SCALE3 ABSPACK algorithm[2]. Structures were solved and refined using Olex2 with the SHELXTL package[3–5].

High-pressure SCXRD

High-pressure single-crystal XRD was taken at room temperature on the piece of single crystal from the crystal characterized at ambient pressure, using a Diacell One20DAC (Almax-easyLab) with 400 μm culet Boehler-Almax anvils and Rigaku XtaLAB Synergy Dualflex diffractometer with a HyPix detector. A stainless-steel gasket (250 μm thick) was pre-indented to 37 μm, and a 200 μm hole was drilled by the electric discharge machine to hold the sample. A 4:1 methanol-ethanol mixture was used as the pressure-transmitting medium to maintain hydrostatic conditions[6]. After loading the sample into the DAC, the pressure was increased in two steps to above 5 GPa, reaching a maximum of 8.5 GPa. The sample remained at pressure above 5.6 GPa for about 10 days. After the pressure was reduced below 5 GPa, measurements at 3.4 and 1.4 GPa were carried out during decompression. Each high-pressure SCXRD measurement took around 40 hours. At least 12 hours were taken for pressure stabilization. The sample was then removed from the DAC and broken into several pieces, one of which was measured immediately. Refinement details and atomic coordination parameters for ambient pressure and representative pressures are summarized in **Tables S1** and **S2.**

Table S1. Crystal structure and refinement data for UTe_2 at ambient pressure (before and after applying pressure) and under 1.4 GPa and 3.4 GPa. Values in parentheses represent estimated standard deviations from the refinements.

	Outside the DAC	Pressure decreases after high pressure treatment	Pressure decreases after high pressure treatment	Outside the DAC after high pressure treatment
Temperature (K)	298.15	297.15	297.15	297.15
Pressure (GPa)	Ambient	3.4	1.4	Ambient
Space Group	*Immm*	*I*4/*mmm*	*I*4/*mmm*	*Immm*
Unit Cell dimensions	a = 4.1642(2) Å<br>b = 6.1406(3) Å<br>c = 13.9869(7) Å	a = 3.9126(15) Å<br>b = 3.9126(15) Å<br>c = 9.912(6) Å	a = 3.9371(10) Å<br>b = 3.9371(10) Å<br>c = 10.145(4) Å	a = 4.1606(8) Å<br>b = 6.1311(12) Å<br>c = 13.974(2) Å
Volume	357.66(3) Å3	151.75(15)	157.25(10)	356.45(11)
Z	4	2	2	4
Density (calculated)	9.160	10.794	10.417	9.191
Absorption coefficient	60.997	71.987	69.467	61.293
F (000)	784	392	392	784
2θ range	10.216 to 81.926	11.208 to 80.516	11.112 to 74.674	7.258 to 67.06
Reflections collected	4248	758	971	2513
Independent reflections	700 [Rint = 0.0613]	128 [Rint = 0.5424]	128 [Rint = 0.5424]	404[Rint = 0.2475]
Data/restraints/parameters	700/0/14	128/0/6	128/0/6	404/0/14
Final R indices	R1 (I>2σ(I)) = 0.0239; wR2 (I > 2σ(I)) = 0. 0540	R1 (I>2σ(I)) = 0.4439; wR2 (I > 2σ(I)) = 0. 6455	R1 (I>2σ(I)) = 0.4885; wR2 (I > 2σ(I)) = 0. 7059	R1 (I>2σ(I)) = 0.1305; wR2 (I > 2σ(I)) = 0.2931
Largest diff. peak and hole	+2.86 e-/Å3, -2.96 e-/Å3	+11.10 e-/Å3, -6.93 e-/Å3	+8.19 e-/Å3 , -6.01 e-/Å3	+12.20 e-/Å3, -10.18 e-/Å3
Goodness-of-fit on F2	1.031	1.504	2.186	1.287

Table S2. Atomic coordinates and equivalent isotropic atomic displacement parameters for UTe_2.

P(GPa)	Atom	Wyck.	x	y	z	Occ.	U_{eq}
**Ambient (before pressure added)**	U	4*j*	1/2	0	0.13513(2)	1	0.009(8)
	Te1	4*g*	0	0.24923(6)	0	1	0.009(9)
	Te2	4*i*	0	0	0.29793(3)	1	0.010(9)
**3.4**	U	2*a*	0	0	0	1	0.079(30)
	Te	2*e*	0	0	0.34400(60)	1	0.070(30)
**1.4**	U	2*a*	0	0	0	1	0.120(20)
	Te	2*e*	0	0	0.33800(40)	1	0.095(14)
**Ambient (After pressure added)**	U	4*j*	1/2	0	0.13540(2)	1	0.017(10)
	Te1	4*g*	0	0.24980(9)	0	1	0.018(10)
	Te2	4*i*	0	0	0.29760(4)	1	0.019(13)

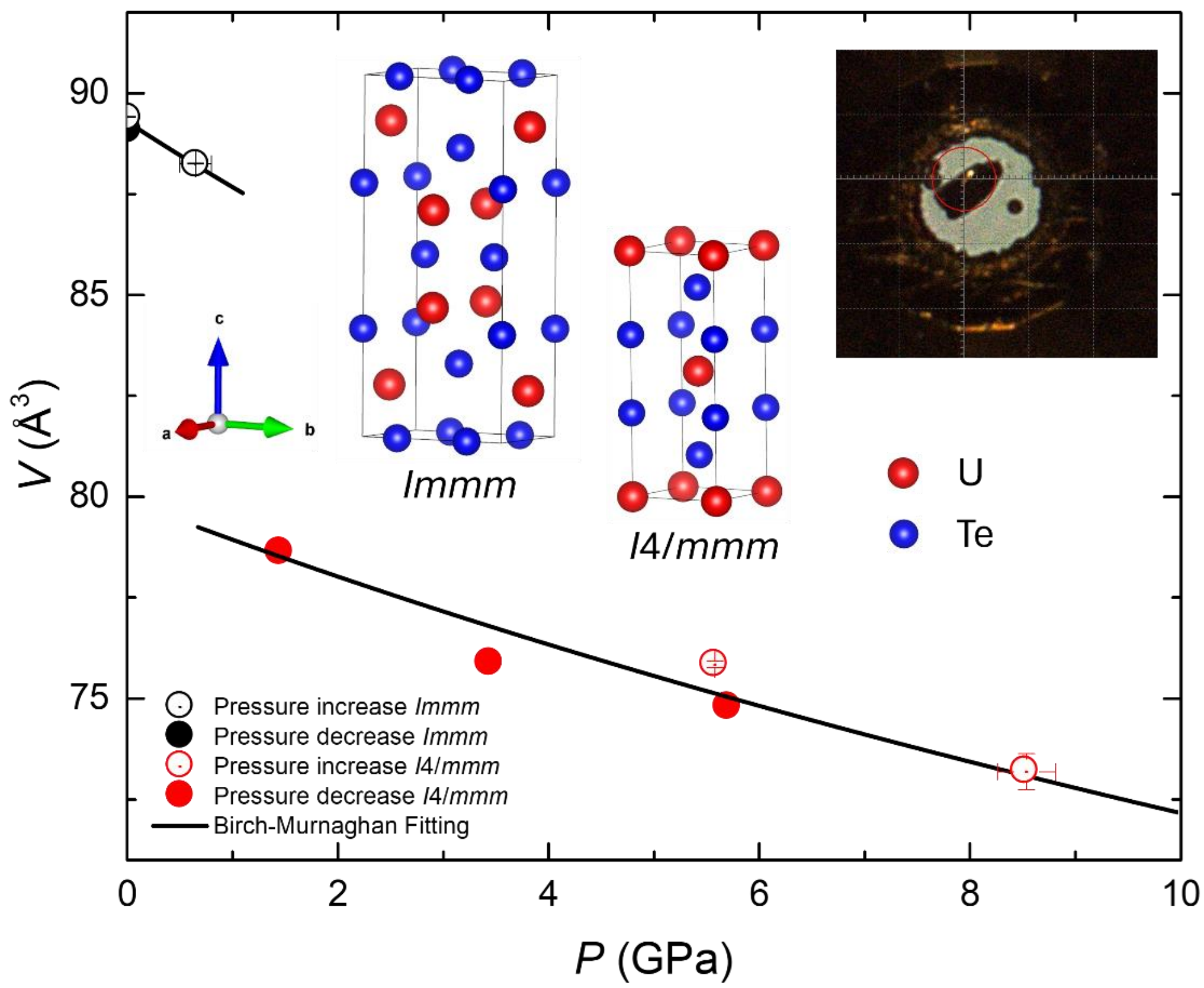

Figure S1. Pressure-dependent evolution of the unit-cell volume (V_{cell}) per chemical formula unit (Z) of UTe_2. V_{cell}/Z versus pressure with a Birch-Murnaghan equation-of-state fit. The top-right inset displays the gasket and sample chamber; the red circle marks a diameter of 0.1 mm. The red or black symbols indicate the *I*4/*mmm* or *Immm* structure of UTe_2, respectively.

Fig. S1 shows the volume changes as the function of the pressure. An UTe_2 single crystal was selected and measured using SCXRD before loaded into DAC. The orthodromic structure (*Immm*) was confirmed at ambient and 0.7 GPa. As the pressure increased to 5.6 GPa, the tetragonal structure (*I*4/*mmm*) was identified. The relatively high R_{int} and R_1 values compared with those of the ambient- and low-pressure data are likely due to the internal strain generated by pressure increase and first order structural transition. The pressure then further increases to 8.5 GPa and maintained for more than 120 hours. The tetragonal structure persisted throughout the entire decompression process. When the pressure was lower than 1.4 GPa, the liquid pressure medium had escaped, and the broken sample was removed from DAC. One piece of broken sample was selected and immediately measured, which shows the recovery of tetragonal *Immm* structure. These results suggest that UTe_2 can keep the tetragonal metastable phases below the structure transition pressure, which is at about 5 GPa during increasing pressure.

AC magnetic susceptibility under pressure

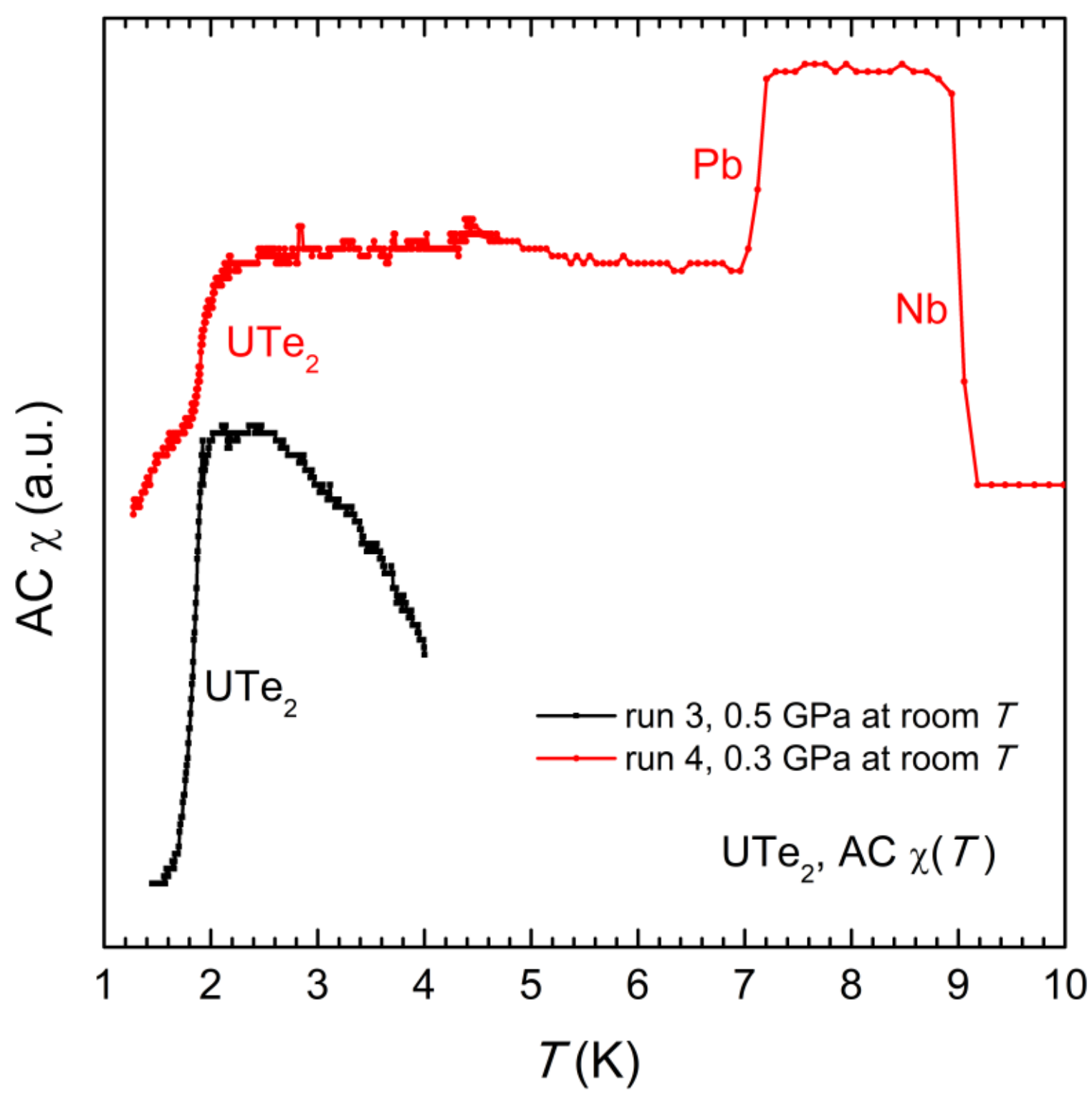

Figure S2. Selected AC magnetic susceptibility (χ real part) curves of the orthorhombic phase of UTe_2 at low temperature and under high pressure. In $\chi(T)$ run 4, a piece of Pb was loaded with UTe_2 in the gasket hole which sat above the secondary coil to calibrate pressures *in situ*. A piece of Nb was loaded into the oppositely wound compensation secondary coil to detect the temperature discrepancy between the thermometer used to measure temperature and the DAC. Superconducting transition curves for UTe_2, Pb, and Nb are shown in the figure.

References

[1] S. Parkin, B. Moezzi, and H. Hope, "XABS2: An empirical absorption correction program," *J. Appl. Crystallogr.* **28**, 53 (1995).

[2] N. Walker and D. Stuart, "An empirical method for correcting diffractometer data for absorption effects," *Acta Crystallogr. A* **39**, 158 (1983).

[3] G. M. Sheldrick, "SHELXT – Integrated space-group and crystal-structure determination," *Acta Crystallogr. A* **71**, 3 (2015).

[4] G. M. Sheldrick, "Crystal structure refinement with SHELXL," *Acta Crystallogr. C* **71**, 3 (2015).

[5] V. Dolomanov, L. J. Bourhis, R. J. Gildea, J. A. K. Howard, and H. Puschmann, "OLEX2: A complete structure solution, refinement and analysis program," *J. Appl. Crystallogr.* **42**, 339 (2009).

[6] X. Chen, H. Lou, Z. Zeng, B. Cheng, X. Zhang, Y. Liu, D. Xu, K. Yang, and Q. Zeng, "Structural transitions of 4:1 methanol–ethanol mixture and silicone oil under high pressure," *Matter Radiat. Extremes* **6**, 035601 (2021).
